# Different Patterns of Lichen Planus in Three Members of One Family

**DOI:** 10.1155/crdm/1177988

**Published:** 2026-02-08

**Authors:** Bahareh Abtahi-Naeini, Maryam Khalili, Fahimeh Shirdel, Mahsa Pourmahdi-Boroujeni

**Affiliations:** ^1^ Pediatric Dermatology Division of Department of Pediatrics, Imam Hossein Children’s Hospital, Isfahan University of Medical Sciences, Isfahan, Iran, mui.ac.ir; ^2^ Skin Diseases and Leishmaniasis Research Center, Isfahan University of Medical Sciences, Isfahan, Iran, mui.ac.ir; ^3^ Pediatric Dermatology Department, Kerman University of Medical Science, Kerman, Iran, kmu.ac.ir; ^4^ Student Research Committee, Isfahan University of Medical Sciences, Isfahan, Iran, mui.ac.ir

**Keywords:** familial Lichen Planus, Frontal Fibrosing Alopecia, Lichen Planopilaris, Lichen Planus, Lichen Planus Pigmentosus

## Abstract

The pathogenesis of Lichen Planus (LP) and its variants, despite many investigative efforts, remains incompletely understood. The occurrence of the disease in siblings suggests a potential role for both genetic and shared environmental factors. Here, three Iranian siblings—one male and two females—who presented with various clinical forms of LP were introduced. Case No. 1 exhibited bilateral facial pigmentation and was diagnosed with Lichen Planus Pigmentosus (LPPigm). Case No. 2 presented with facial pigmentation accompanied by facial papules and was diagnosed with LPPigm and Lichen Planopilaris (LPP). Case No.3 had frontotemporal hairline recession and eyebrow sparsening and was diagnosed with Frontal Fibrosing Alopecia (FFA). Histopathological examination confirmed the diagnoses in all three patients. Patients were treated with an individualized plan that included sunscreen use, potent topical corticosteroids, topical pimecrolimus, minoxidil, and systemic finasteride. To our knowledge, no other family in the literature has been reported to have such a wide range of LP variants. This series underscores the need for further research into genetic and environmental factors contributing to the development of LP and its variants.

## 1. Introduction

Lichen Planus (LP) encompasses several variants, including Lichen Planopilaris (LPP), Lichen Planus Pigmentosus (LPPigm), and Frontal Fibrosing Alopecia (FFA). Despite the increasing number of reported cases, the exact causes of FFA and LPP remain unclear, although both are believed to have an immune‐mediated component [1–4].

The occurrence of familial FFA cases and the fact that FFA usually affects older individuals suggest that both genetic and environmental factors contribute to disease pathogenesis. Recent genetic investigations have provided some insights into potential pathogenic mechanisms; however, further investigation is needed for a more comprehensive understanding [5, 6].

**FIGURE 1:**
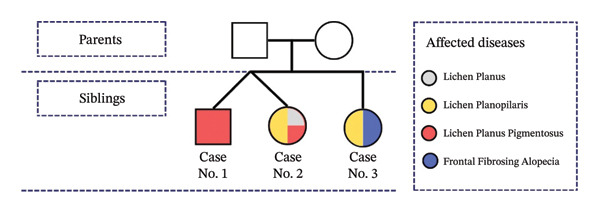
Pedigree diagram of a family affected by different clinical types of Lichen Planus.

Herein, we present three siblings from an Iranian family with different LP variants. The patients were between 50 and 60 years and had no history of other underlying diseases (Figure 1).

## 2. Case Presentation

### 2.1. Case 1

**FIGURE 2:**
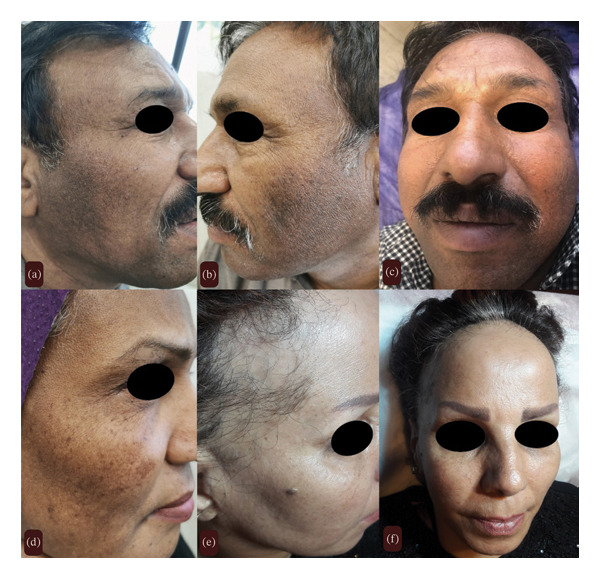
Three siblings with multivariant forms of Lichen Planus: (a–c) 56‐year‐old man with bilateral facial pigmentation and multiple round‐to‐oval blue‐gray macules with the final diagnosis of Lichen Planus Pigmentosus. (d) 56‐year‐old woman with facial papules and facial pigmentation with the final diagnosis of Lichen Planus Pigmentosus and Lichen Planopilaris. (e, f) 52‐year‐old woman with frontotemporal hairline recession, eyebrow loss, and facial papules with the final diagnosis of frontal fibrosing alopecia and Lichen Planus.

A 56‐year‐old male, Fitzpatrick skin Type 4, was referred to an outpatient dermatology clinic with a chief complaint of bilateral facial pigmentation. Pigmentation began about 2 years prior, without preceding facial erythema or pruritus. On facial examination, symmetrical round‐to‐oval blue‐gray macules and patches were observed on the lateral aspect of cheeks and beard (Figure 2). There was no other site of involvement or any facial papules.

Since childhood, he has had frequent sun exposure without sufficient sun protection. He was a smoker but had no other specific underlying condition. The medication history was unremarkable. Initially, with a diagnosis of melasma, multiple topical whitening treatments, such as hydroquinone, were prescribed. However, there was no significant clinical response, and the pigmentation worsened.

**FIGURE 3:**
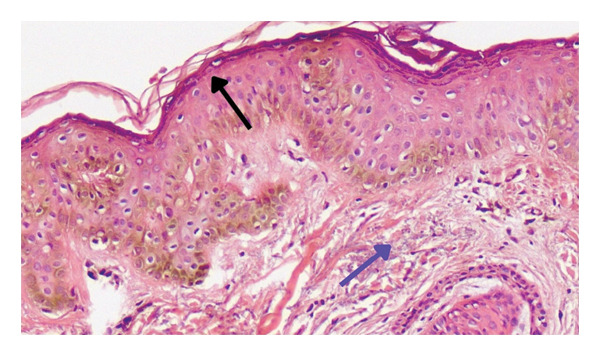
Skin biopsy of a 56‐year‐old man with Lichen Planus Pigmentosa: hyperpigmentation of the basal layer (black arrow), focal hydropic change, basket‐wave orthokeratosis, hypergranulosis, slight acanthosis, solar degeneration (blue arrow), and melanin incontinent (hematoxylin and eosin; magnification: × 10).

Therefore, with clinical differential diagnoses of LPPigm, melasma, drug‐induced hyperpigmentation, postinflammatory hyperpigmentation, and pigmented contact dermatitis, a 4‐mm punch biopsy was obtained from the face. Histopathological evaluation showed basket‐wave orthokeratosis, hypergranulosis, slight acanthosis, pigmentation of the basal layer, focal hydropic change, solar degeneration, and melanin incontinence, compatible with the diagnosis of facial LPPigm (Figure 3).

According to the diagnosis, treatment with topical potent corticosteroids was started and continued periodically. Pimecrolimus cream was also prescribed twice daily for application to the pigmented area. The patient was also reminded of the importance of frequent sunscreen use. At the 9‐month follow‐up, the pigmentation area remained stable, and disease progression had stopped.

### 2.2. Case 2

A 56‐year‐old postmenopausal female patient with Fitzpatrick Type 4 skin, who was the twin sister of Case No. 1, presented with a chief complaint of facial pigmentation. Pigmentation began about 5 years prior, and despite using multiple topical bleaching agents and undergoing laser‐assisted melasma treatment, no clinical response was observed, similar to her brother. Additionally, she had scalp erythema and pruritus, which began 2 years prior and were associated with facial monomorphic papules (Figure 1).

**FIGURE 4:**
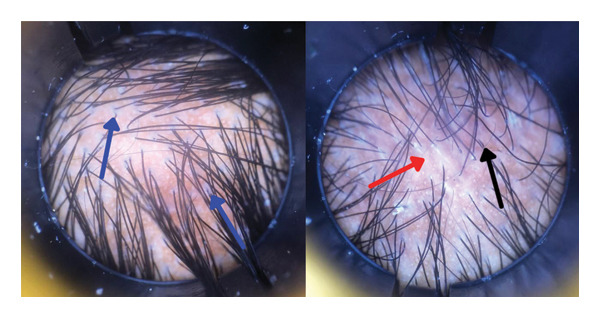
Trichoscopy examination of a 56‐year‐old woman with Lichen Planus Pigmentosus and Lichen Planopilaris: perifollicular hyperkeratosis and white scale (blue arrow), perifollicular erythema (black arrow), and a small area of scar formation (red arrow).

Trichoscopy examination revealed perifollicular hyperkeratosis, perifollicular erythema, and a small area of scar formation (Figure 4). There was no eyelash or eyebrow involvement. The histopathological examination of the facial pigmentation was compatible with the diagnosis of LPPigm. Therefore, the patient was considered LPPigm, associated with LPP (based on the scalp involvement) and a facial LP papule. The treatment was similar to his brother’s, with potent topical corticosteroids and pimecrolimus cream. During follow‐up, perifollicular hyperkeratosis and erythema gradually decreased, and the facial papules resolved. For the pigmented areas, tranexamic acid injections were administered, resulting in approximately 50% improvement in pigmentation. At the 9‐month follow‐up, the condition had entered remission.

### 2.3. Case 3

A 52‐year‐old postmenopausal woman with Fitzpatrick Type 3 skin, and the sister of the other two patients, presented with a 5‐year history of symmetric, band‐like frontotemporal hairline recession and partial eyebrow sparsening. The affected scalp appeared shiny and smooth, and the hair pull test was negative. Dermoscopic examination of the frontotemporal region revealed a marked reduction in follicular ostia along with perifollicular erythema, scaling, and hyperkeratosis (Figure 2). She also had facial papules; however, examination of the oral and genital mucosae, nails, and the rest of the body showed no abnormalities.

A skin biopsy confirmed the diagnosis of LPP. Based on the clinical presentation, trichoscopic findings, and histopathology, the patient was diagnosed with FFA associated with LPP. She was treated with finasteride (5 mg/day) and topical minoxidil. Periodic topical clobetasol and pimecrolimus cream were also prescribed for the facial papules. At the 9‐month follow‐up, alopecia progression had stopped with no hair growth in the frontotemporal region, and the condition had entered remission.

Notably, both parents passed away years ago. Given the absence of detailed medical examinations, their health status and the presence of any lichenoid mucocutaneous condition cannot be definitively determined. Additionally, there were no more siblings in their family.

## 3. Discussion

Here, we presented three siblings who were diagnosed with various forms of LP, including FFA, LPP, and LPPigm. In Case No. 1, LPPigm manifested as bilateral, symmetrical blue‐gray macules and patches confined to the face, without papules or scalp involvement. In Case No. 2, LPPigm manifested as bilateral facial pigmentation. Also, Case No. 2 presented facial papules compatible with the diagnosis of LP, as well as LPP of the scalp, characterized by perifollicular erythema, hyperkeratosis, and scarring alopecia on trichoscopy. Case No. 3 presented with the features of FFA, including band‐like frontotemporal hairline recession and scalp LPP, similar to Case No. 2. Although the coexistence of these diseases has been reported in one patient [7], a family with such variants of LP has not been reported so far.

The occurrence of familial LP with multivariant forms suggests a possible role for both genetic and environmental factors in the etiopathogenesis of the LP spectrum. Upon the literature search, reports of eight families with multivariant forms of LP, emphasizing FFA and LPP, were noted. Table 1 presents clinical evaluations, diagnoses, other underlying diseases, HLA typing, and treatments of these families (Table 1).

**TABLE TABLE​ 1:** Clinical, demographic, histological, HLA‐typing, and treatment data of patients with familial Lichen Planus consisting of various forms of LPP, FFA, and LPPigm including literature review.

Author/year	Ethnicity	Gender–relativity	Age at diagnose	Clinical evaluation and underlying conditions	Diagnosis	HLA typing	Treatments
Dlova et al. [1] 2013	Italian Caucasian	M–Brother	62	Eyebrow loss, body hair loss FFA	Clinically + Biopsy Main histologic findings: erythema and scaling	Not Evaluated	NM
		F–Sister	59	Eyebrow Loss, Body Hair Loss FFA, LPP			NM

Rivas et al. [11] 2015	Spain	F–Mother	67	Decreased hair density in male pattern, Slight eyebrows alopecia FFA Chronic Autoimmune Gastric Atrophy	Clinically + Biopsy Main Histologic Findings: Follicular destruction, Perifollicular lymphoid cell infiltration	HLA DR B1 ^∗^ 04, 13 HLA DQ B1 ^∗^ 03: 02, 06	Minoxidil, Retinoic Acid, Betamethasone, Dipropionate
		F–Daughter	38	Partial Occipital Patches of Cicatricial Alopecia LPP	Clinically + Biopsy Main Histologic Findings: Follicular destruction with fibrosis replacement, Laminated fibrotic rims around follicles, Perifollicular lymphoid cell infiltration		Tacrolimus Ointment

Namazi et al. [13] 2018	Iranian	F–Mother & Sister	50	Facial Papules, Vellus Hair LPP	Clinically + Biopsy Main histologic findings: NM	Not Evaluated	Hydroxychloroquine Isotretinoin
		F–Sister	41	Eyebrows alopecia, facial papules FFA, LPP	Clinically + Biopsy main histologic findings: Perifollicular lymphoid cell infiltrate, Follicular destruction, Mild acanthosis, Focal dermal fibrosis perifollicular mucin deposition		Hydroxychloroquine, cyclosporine, topical corticosteroids, mycophenolate mofetil, laser therapy, and finasteride Isotretinoin
		F–Sister	35	Facial papules, vellus hair LPP	Clinically + Biopsy Main histologic findings: NM		Isotretinoin
		M–Son	30	Facial papules, bilateral beard alopecia LPP on the beard	Clinically + Biopsy Main histologic findings: NM		Hydroxychloroquine Isotretinoin

Porriño‐Bustamante et al. [5] 2019	Caucasian	M–Brother	44	Partial eyelashes alopecia, facial papules, total hair loss of beard, lower limbs, and forearms, Partial hair loss of axilla and pubis	Clinically + Biopsy Main histologic findings: Perifollicular lymphoid cell infiltrate, interface dermatitis, concentric perifollicular fibrosis, follicular destruction, loss and atrophy of sebaceous glands	Not Evaluated	NM
		M–Brother	46	Partial eyelashes alopecia, facial papules, partial hair loss of beard, and all limbs, patches of scaring alopecia FFA, LPP			NM

Rocha et al. [6] 2020	Brazilian	F–Sister	67	Eyebrow and eyelashes loss, body hair loss Diffused FFA, LPPigm	Clinically	Not Evaluated	NM
		F–Sister	64	Eyelashes and partial eyebrow loss Diffused and patchy FFA	Clinically		NM
		F–Sister	62	Eyelashes loss, body hair loss Diffused FFA	Clinically		NM
		F–Sister	61	Facial papules, eyelashes and partial eyebrow loss, body hair loss Diffused FFA Allergic rhinitis	Clinically		NM
		F–Sister	52	Facial papules, eyebrow loss, body hair loss Diffused FFA	Clinically		NM
		F–Sister	51	Facial papules, eyelashes and eyebrow loss Diffused FFA	Clinically		NM

Ocampo‐Garza et al. [3] 2021	Spain	F–Mother	72	Eyelashes and eyebrow loss, pubis hair loss, depressed frontal veins FFA Rheumatoid arthritis, surgical menopausal	Clinically + Biopsy Main histologic findings: NM	Not Evaluated	NM
		F–Daughter	51	Facial papules, eyebrow loss, limb’s hair loss LPPigm	Clinically + Biopsy Main histologic findings: NM		NM
	Spain	F–Mother	58	Facial papules, Retroauricular hairline recession, eyebrow loss, limbs and axilla hair loss, depressed frontal veins LPPigm, FFA DM, HTN, and postmenopausal	Clinically + Biopsy Main histologic findings: NM	Not evaluated	NM
		F–Daughter	39	Dermoscopic characteristic of FFA Autoimmune hypothyroidism	Clinically + Biopsy Main histologic findings: NM		NM

Our cases	Iranian	M–Brother (Twin)	56	Bilateral facial pigmentation Multiple round‐to‐oval blue‐gray macules Facial erythema LPPigm	Clinically + Biopsy Main histologic findings: Basket‐wave orthokeratosis, hypergranulosis, slight acanthosis, pigmentation of the basal layer, focal hydropic change, solar degeneration, melanin incontinence	Not evaluated	Topical corticosteroids Topical pimecrolimus
		F–Sister (Twin)	56	Facial pigmentation (LPPigm) Facial papules (LP) LPP	Clinically + Biopsy Main histologic findings: Perifollicular hyperkeratosis, perifollicular erythema, scar formation		Topical corticosteroids Topical pimecrolimus
		F–Sister	52	FFA Eyebrow loss Negative hair pull test Facial papules (LP)	Clinically + Biopsy Main histologic findings: Perifollicular erythema, perifollicular hyperkeratosis, follicular destruction		Topical corticosteroids Topical pimecrolimus Finasteride Topical minoxidil

*Note:* LPP, Lichen Planopilaris, LPPigm: Lichen Planus Pigmentosus, HTN: hypertension.

Abbreviations: DIF, direct immune fluorescence; DM, diabetes mellitus; FFA, Frontal Fibrosing Alopecia; HLA, human leukocyte antigen; LP, Lichen Planus; NM, not mentioned.

FFA presents with a gradual frontotemporal hairline recession accompanied by eyebrow alopecia. In contrast, classic LPP shows irregular areas of alopecia in the vertex region without a characteristic band‐like distribution seen in FFA [2]. FFA and LPP are generally considered different clinical presentations within the same disease spectrum. The findings in both conditions demonstrate follicular destruction with a lymphocytic infiltrate targeting the isthmus and infundibulum of the hair follicles, apoptotic cells within the external root sheath, and concentric perifollicular fibrosis resulting in scarring alopecia. Notably, FFA tends to show less follicular inflammation and a higher rate of apoptotic cells. On the other hand, in some LPP cases, the inflammatory infiltrate may extend into the interfollicular epidermis. Direct immunofluorescence (DIF) study of the FFA biopsies typically shows no specific findings. In contrast, LPP biopsies demonstrate globular deposits, cytoid bodies (usually IgM), as well as irregular fibrinogen and complement deposition within the papillary dermis adjacent to the follicular epithelium [8].

LPPigm presents as acquired asymmetric pigmented macules, predominantly affecting sun‐exposed areas. It commonly affects dark‐skinned patients, particularly those from India and the Middle East. In Table 1, the majority of the described cases were Caucasian patients (Table 1). Further investigations are needed to clarify the role of ethnicity as a whole genetic component. Key histopathological features include hyperkeratosis, basal cell vacuolar degeneration, epidermal thinning, perivascular infiltrates, pigment incontinence, and band‐like lichenoid infiltrates [7, 9]. In the current study, Cases No. 1 and No. 2—both with relatively darker skin—had LPPigm, and Case No. 2 also presented with LPP and LP. Case No. 3 exhibited FFA without the features of LPPigm. Nevertheless, the coexistence of FFA and LPPigm has been reported previously [2].

Studies show that HLA‐DR1 plays a significant role in the pathogenesis of LP and Lassueur–Graham–Little–Piccardi syndrome. Nevertheless, such concurrency is not reported in FFA. HLA B^∗^07:02 is one of the most prevalent alleles in FFA cases [6]. Table 1 represents the HLA findings of the included families with multivariant forms of LP (Table 1). Unfortunately, due to resource limitations, our cases did not undergo any genome typing.

Hormonal factors are other predisposing factors that may contribute to the pathogenesis of FFA [10]. The predominance of FFA among elderly postmenopausal women, as demonstrated in Table 1, supports this hypothesis (Table 1). In our cases, Cases No.2 and No.3 were postmenopausal women. Moreover, finasteride—a 5‐alpha‐reductase inhibitor—has been shown in some cases to stop disease progression [11]. Therefore, Case No. 3 planned to receive finasteride, which successfully stopped disease progression.

There are reports of autoimmune diseases occurring concurrently with FFA and LPP, including vitiligo, autoimmune thyroid disorders, and autoimmune gastritis. Chronic diseases associated with immune dysregulation may also play an important role [3, 10]. In addition, several exogenous factors have been proposed to play a role in the pathogenesis of LP and its variants, including Hepatitis C virus, human immunodeficiency virus, herpes simplex Virus 2*, Helicobacter pylori*, human papillomavirus, and syphilis [9, 12, 13]. In our cases, the coexistence of LP variants and no other autoimmune or underlying systemic disease was identified.

In conclusion, this report highlights the possibility of familial LP with multivariant forms, suggesting the involvement of genetic and environmental factors in the etiopathogenesis of the disease.

## Author Contributions

Bahareh Abtahi‐Naeini: conceptualization, supervision, project administration, and writing–review and editing.

Maryam Khalili: conceptualization, resources, validation, and writing–review and editing.

Fahimeh Shirdel: methodology, investigation, and writing–review and editing.

Mahsa Pourmahdi‐Boroujeni: investigation, visualization, writing–original draft, and writing–review and editing.

All authors have revised the manuscript critically for important intellectual content and confirmed the accuracy or integrity of any parts of the work.

## Funding

The authors received no specific funding for this work.

## Disclosure

We declare that none of the authors are employed by a government agency that has a primary function other than research and/or education. None of the authors have an official representative on behalf of the government. All authors have read and approved the content of the manuscript.

## Ethics Statement

The Ethics Committee of the Isfahan University of Medical Sciences in Isfahan, Iran, approved this report’s ethical content (IR.ARI.MUI.REC.1402.195). Participation in the study was contingent upon signing a written informed consent form signed by all the patients. Written informed consent was obtained from all three patients for publication of the details of their medical case and any accompanying images.

## Conflicts of Interest

The authors declare no conflicts of interest.

## Data Availability

Upon written request, the corresponding author will provide the data used to support the findings and conclusions.
